# The Song of the Iron Pill

**Published:** 1887-04-23

**Authors:** 


### THE SONG OF THE IRON PILL.

Doctor O'Grady attended "my Lady,"
Who had in her ears a loud ringin'.
"Bedad thin my lady," said Dr. O'Grady,
"It's me'silf will soon cure that same singin'!"

"You must try this small pill—'Ferri sulph. pot. carb.'—till
You can take six a day afther 'atin:
Take it on for six weeks: then a rest it bespeaks:
Afther that you'll the 'course' be repatin'.

"It will strengthen the chest, set the timper at rest,
And bid the head-noises be packin';
It will bring just a hint of the finest rouge tint
On your cheeks, now in roses so lackin'.

"Of heart's palpitatin' it steadies the batin';
To shortness of breath it's confusion;
If you'll use it quite fair I'll make bould to declare
Never more you'll call physic 'delusion.'"

